# Neoadjuvant and Adjuvant Treatments Compared to Concurrent Chemoradiotherapy for Patients With Locally Advanced Cervical Cancer: A Bayesian Network Meta-Analysis

**DOI:** 10.3389/fonc.2022.745522

**Published:** 2022-03-16

**Authors:** Yunfeng Qiao, Huijun Li, Bing Peng

**Affiliations:** ^1^ Health Science Center, Yangtze University, Jingzhou, China; ^2^ Cancer Center, Renmin Hospital of Wuhan University, Wuhan, China; ^3^ Department of Oncology, The Second People’s Hospital of Jingmen, Jingmen, China

**Keywords:** chemoradiotherapy, chemotherapy, radiotherapy, network meta-analysis, locally advanced cervical cancer

## Abstract

**Aim:**

This study aimed to identify the most effective treatment mode for locally advanced cervical cancer (LACC) by adopting a network meta-analysis (NMA).

**Methods:**

Randomized controlled trials about treatments were retrieved from PubMed, Medline and Embase. Odds ratios (OR) of overall survival (OS) and progression-free survival (PFS) were calculated by synthesizing direct and indirect evidence to rank the efficacy of nine treatments. Consistency was assessed by node-splitting method. Begg’s test was performed to evaluate publication bias. The surface under cumulative ranking curve (SUCRA) was also used in this NMA.

**Results:**

A total of 24 eligible randomized controlled trials with 6,636 patients were included in our NMA. These trials compared a total of nine different regimens: radiotherapy (RT) alone, surgery, RT plus adjuvant chemotherapy (CT), concurrent chemoradiotherapy (CCRT), neoadjuvant CT plus CCRT, CCRT plus adjuvant CT, neoadjuvant CT, RT, CCRT plus surgery. Among those therapeutic modalities, we found that the two interventions with the highest SUCRA for OS and PFS were CCRT and CCRT plus adjuvant CT, respectively. ORs and 95% confidence interval (CI) for the two best strategies were CCRT versus CCRT plus adjuvant CT (OR, 0.84; 95% CI, 0.53–1.31) for OS, CCRT plus adjuvant CT versus CCRT (OR, 0.60; 95% CI, 0.38–0.96) for PFS.

**Conclusions:**

This NMA supported that CCRT and CCRT plus adjuvant CT are likely to be the most optimal treatments in terms of both OS and PFS for LACC. Future studies should focus on comparing CCRT and CCRT plus adjuvant CT in the treatment of LACC.

**Systematic Review Registration:**

PROSPERO, CRD42019147920.

## Introduction

Cervical cancer remains the fourth most common and lethal female malignancy worldwide, with an estimated 569,847 new cases and 311,365 deaths worldwide reported in 2018 (1). Currently, the average 5-year survival rate of cervical cancer has reached 66% in developed countries, yet less than half of patients from developing countries could live longer than 5 years (2, 3). Several controversies still exist for the optional management of locally advanced cervical cancer [LACC; International Federation of Gynecology and Obstetrics (FIGO) stages IB2-IVA], which represents almost 60% of all diagnosed cervical cancers, with a 3-year OS about 81% for stage IB2, 51% for stage IIIB and 28% for stage IVA, respectively (4).

Many trials have shown that concurrent chemoradiotherapy (CCRT) reduces the risk of death for LACC by 30 to 50% compared with radiotherapy (RT) alone (5–9). Based on these data, the National Cancer Institute suggested that strong consideration should be given to using CCRT instead of RT alone for LACC (10). Surgery is still a common treatment option, and neoadjuvant chemotherapy (CT) before surgery has been shown to improve survival in selected LACC patients (11, 12). Although the approach of neoadjuvant CT plus surgery lacks adequate evidence, it is practiced in many parts of the world (13, 14). The role of adjuvant CT after CCRT for LACC has also been explored in many studies (15–18). However, there is much debate because four randomized controlled trials of adjuvant CT after CCRT have inconsistent data when compared with CCRT (15–18). Presently, although more interests are focused on neoadjuvant CT before CCRT (19–21), there is only one phase II research about addressing this strategy compared with CCRT (21). With lots of neoadjuvant and adjuvant therapies, the optimal strategy for the management of LACC remains to be characterized.

Network meta-analyses (NMA) provide an opportunity to perform direct and indirect treatment comparisons among randomized studies without breaking randomization, as long as specific assumptions are fulfilled (22). Through indirect measures, NMA enables estimation of comparative efficacy for interventions that have not been investigated in direct head-to-head randomized trials (e.g., comparison of the treatment A vs C, using data from trials comparing A vs B and B vs C). Thus, we employed Bayesian NMA to compare the outcomes of different treatment modalities for LACC.

## Materials and Methods

### Search Strategy

We carried out a systematic search of available literature and results which were reported in adherence to the preferred reporting items for systematic reviews and meta-analyses (PRISMA) guidelines (23). A prospective protocol was created in advance and uploaded to the PROSPERO online platform, with the registration number CRD42019147920. PubMed, Medline and Embase databases were searched for randomized controlled trials, using different combination of the following terms: (“cervical cancer” or “cervix cancer”) AND (‘‘neoadjuvant” or “adjuvant’’) AND one of the following terms per time: “chemotherapy”, “radiotherapy” or “radiation”, “chemoradiotherapy”, “radiochemotherapy” or “chemoradiation”, “surgery’’ or ‘‘hysterectomy”. The last search was performed on September 1, 2019. Only published, full-length articles were included. All these works mentioned above were done by first two reviewers independently, while the last author acted as referee in case of controversies.

In general, one study would be adopted if it satisfied all the following criteria: (1) prospective randomized controlled trials in previously untreated LACC; (2) at least one of interventions mentioned above should be used to treat the cervix cancer of patients; (3) the endpoints included either overall survival (OS) or progression-free survival (PFS). Studies that belong to any one of categories below would be excluded: (1) duplicate studies; (2) single arm trials; (3) letters, reviews, and meta-analysis.

### Statistical Analysis

The study endpoints were OS and PFS and the outcome measure was the odds ratios (OR) with its 95% confidence interval (CI). Three-year OS and PFS were collected as the primary outcomes, since 1-year OS and PFS were a short-term evaluation index which showed no significant difference in most cases, and many studies did not provide 5-year OS and PFS. If the exact number of deaths or living patients was not reported, it would be estimated directly from the Kaplan–Meier survival curve wherever feasible. Treatment regimens in selected randomized controlled trials were first compared in traditional pairwise meta-analyses using a random-effects model. *P*-values <0.05 (2-sided) were considered statistically significant. All direct comparison statistical analyses were performed using the Review Manager software (RevMan v 5.3.5).

Compared with traditional pairwise meta-analyses, the key strength of network meta-analysis is transitivity (24). That is, an indirect estimate of the treatment A vs. C can be acquired by comparing trials of A vs. B and B vs. C. Another key assumption underlying the NMA is similarity. To examine similarity, the population, intervention, comparison, and outcome (PICO) technique is used (25). In order to evaluate the relative effectiveness of the nine treatments, Bayesian NMA was adopted to integrate the comparison of network. Considering the included studies might differ in population characteristics and treatments implementation effect sizes. We allowed varying true effects among studies, which rendered the random effects model to be applied in this NMA (26). The results of our analysis were presented by league table. Node-split models were fit to evaluate inconsistency among comparisons, and *P <*0.05 indicated significant inconsistency (27). Our NMA also provided a ranking probability curve of each treatment to assess the probability of each treatment to be the best, second best, and so on. The surface under the cumulative ranking (SUCRA) lines for each treatment, which equaled 1 when a treatment was certain to be the best and 0 when a treatment was certain to be the worst, were used for treatment ranking.

In addition, we used the Jadad scale (**Table S1**) to independently evaluate the quality of the study included in our NMA. Statistical analysis and graph generation were performed with Stata 15.0 (StataCorp, College Station, TX) (28).

### Patient and Public Involvement

Patients and the public were not involved in the design or conduct of the study.

## Results

### Literature Search

**Figure 1:**
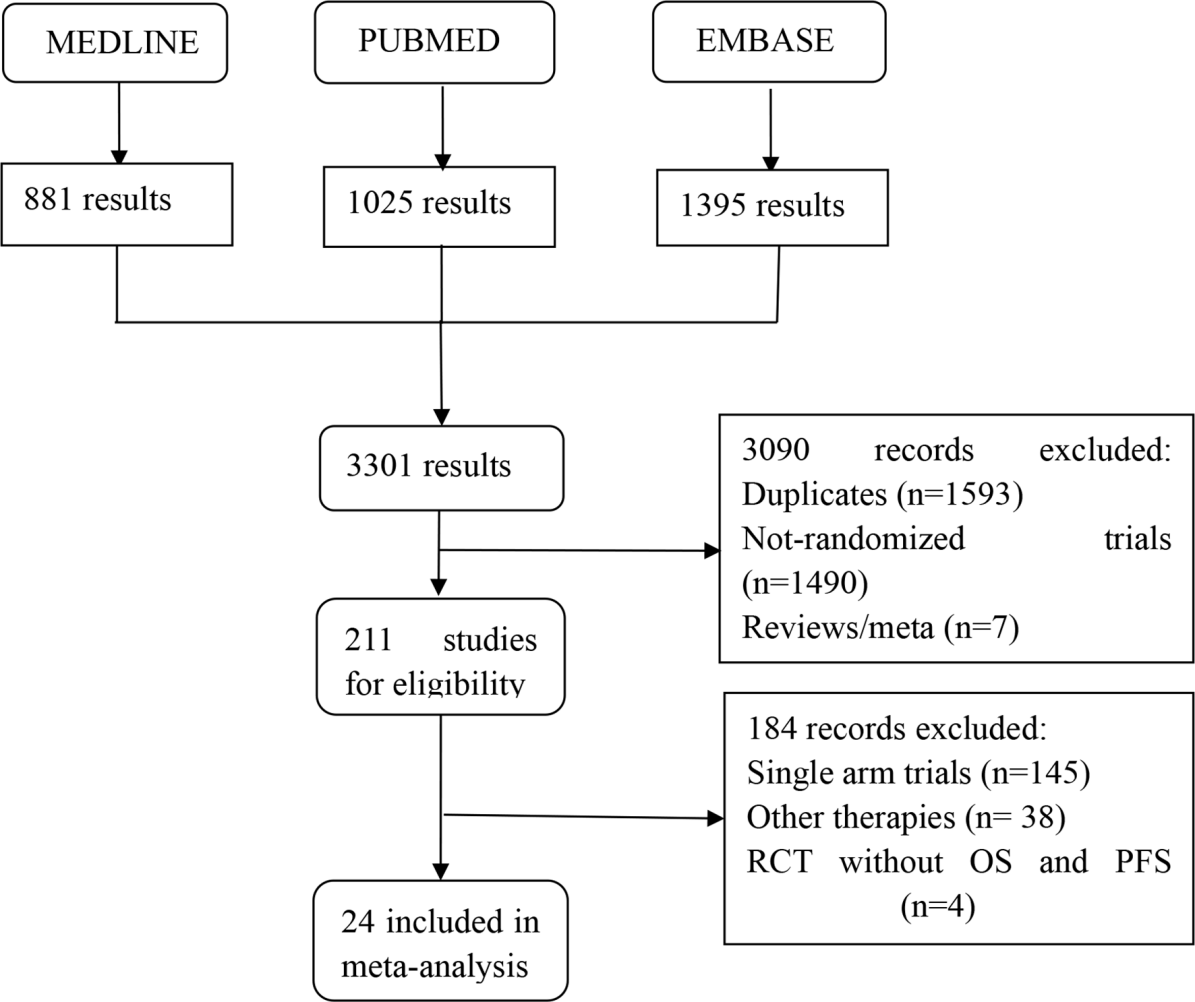
Flow diagram showing inclusion and exclusion of studies according to the PRISMA guidelines.

**Figure 2:**
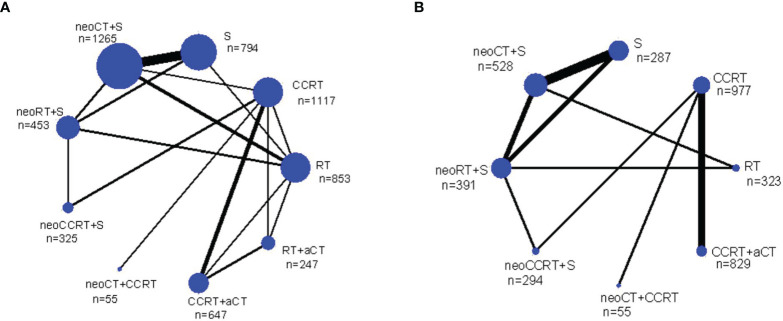
Network diagrams of the overall survival **(A)** and progression free survival **(B)** for the multimodality treatments included in the analysis. Each link represents at least 1 study and the widths of each link are proportional to the number of studies comparing the particular arms. The size of each node is proportional to the total sample size. a, adjuvant; neo, neoadjuvant; n, number of patients; CCRT, chemoradiotherapy; CT, chemotherapy; RT, radiotherapy; S, surgery.

**Figure 1** summarizes the selection process and reasons for exclusion. Twenty-four studies, published between 1987 and 2019 and a total of 6,636 LACC patients were finally included in the meta-analysis (5, 15–18, 21, 29–46). Among them, 1,265 received neoadjuvant CT plus surgery, 453 received neoadjuvant RT plus surgery, 325 received neoadjuvant CCRT plus surgery, 853 received RT alone, 247 received RT plus adjuvant CT, 794 received surgery alone, 1,557 received CCRT alone, 55 received neoadjuvant CT plus CCRT, and 1,087 received CCRT plus adjuvant CT. **Table 1** provides details of the treatment modalities used in each study included in this meta-analysis. Treatment network is shown in **Figure 2**.

**Table 1:** Summary of the studies included in the meta-analysis.

Study	Country	Phase	Design	LACC(%)	SCC(%)	Median age (years)	Stage	Type	Size/Completed size	Outcomes
Keys (5)	USA	III	RCT	100	82	–	IB2	neoRT + S	186	OS/PFS
				100	80	–		neoCCRT + S	183	
Lorvidhay (15)	Thailand	III	RCT	100	89.3	50	IIB–IVA	RT	242	OS
				100	91	49		RT + aCT	221	
				100	88.4	48		CCRT	233	
				100	90	50		CCRT + aCT	230	
Duenas (16)	–	III	RCT	100	93.4	45 (22–68)	IIB–IVA	CCRT + aCT	259	OS/PFS
				100	94.1	46 (18–70)		CCRT	256	
Tang (17)	China	–	RCT	100	0	58.7	IIB–IVA	CCRT	440	PFS
				100	0	53.6		CCRT + aCT	440	
Tangjitgamol (18)	Thailand	III	RCT	100	76	50 (26–68)	IIB–IVA	CCRT	129	OS/PFS
				100	76.9	49 (23–68)		CCRT + aCT	130	
Costa (21)	Brazil	II	RCT	100	87.2	48 (22–69)	IIB–IVA	neoCT + CCRT	55	OS/PFS
				100	88.4	45 (20–67)		CCRT	52	
Gupta (29)	India	III	RCT	100	100	50 (27–65)	IB2–IIB	neoCT + S	316	OS
				100	100	48 (26–65)		CCRT	317	
Wang (30)	China	–	RCT	100	100	–	II–III	RT + aCT	26	OS
				100	100	–		CCRT + aCT	28	
Morice (31)	France	III	RCT	100	90	45 (24–69)	IB2, II	neoCCRT + S	31	OS
				100	80	44 (28–69)		CCRT	30	
Cetina (32)	Mexico	III	RCT	100	90.1	45 (25–62)	IB2–IIB	neoCCRT + S	111	OS/PFS
				100	83	44 (23–66)		CCRT	100	
Chang (33)	Taiwan	III	RCT	100	91	46 (33–69)	IB2, IIA2	neoCT + S	68	OS
				100	88	47 (32–70)		RT	52	
Benedetti (34)	Italy	III	RCT	100	100	49 (25–70)	IB2–III	neoCT + S	210	OS/PFS
				100	100	52 (28–69)		RT	199	
Yamauchi (35)	Japan	–	RCT	100	100	53.2	IIIB	neoCT + S	20	OS
				100	100	59.9		RT	22	
Perez (36)	USA	–	RCT	100	–	–	IB–IIA	neoRT + S	62	OS
				100	–	–		RT	56	
Keys (37)	USA	III	RCT	100	86	–	IB2	neoRT + S	132	OS/PFS
				100	86	–		RT	124	
Landoni (38)	Italy	–	RCT	37.1	81.2	–	IB1–IIA2	S	170	OS
				40.7	85	–		RT	158	
Wen (39)	China	II	RCT	100	87.1	45.0	IB2–IIA	neoRT + S	31	OS/PFS
				100	93.4	45.2		neoCT + S	61	
				100	93.5	45.7		S	31	
Li (40)	China	II	RCT	100	100	40	IB2–IIA	neoRT + S	42	OS/PFS
				100	100	43		S	46	
				100	100	42		neoCT + S	45	
Sardi (41)	Argentina	III	RCT	59.8	100	39 (23–68)	IB1, IB2	neoCT + S	102	OS
				54.4	100	41 (24–69)		S	103	
Cai (42)	China	III	RCT	70.8	76.9	45.6	IB1, IB2	neoCT + S	52	OS
				55.6	72.2	44.8		S	54	
Eddy (43)	USA	III	RCT	100	78	–	IB2	neoCT + S	145	OS/PFS
				100	77	–		S	143	
Chen (44)	China	III	RCT	100	83.3	–	IB2–IIB	neoCT + S	72	OS
				100	81.4	–		S	70	
Katsumata (45)	Japan	III	RCT	100	100	47 (28–70)	IB2–IIB	neoCT + S	67	OS/PFS
				100	99	46 (22–67)		S	67	
Yang (46)	China	–	RCT	100	83.5	47 (23–66)	IB2–IIB	neoCT + S	107	OS
				100	80.9	48 (26–68)		S	110	

LACC, locally advanced cervical cancer; SCC, squamous cell carcinoma; %, percentage; RCT, randomized clinical trial; a, adjuvant; neo, neoadjuvant; CCRT, chemoradiotherapy; CT, chemotherapy; RT, radiotherapy; S, surgery; OS, overall survival; PFS, progression free survival.

### Direct Meta-Analysis

Direct comparison in meta-analysis of efficacy was feasible for 3-year OS in the following: neoadjuvant CT plus surgery versus surgery (8 trials, n = 1,275), neoadjuvant CT plus surgery versus RT (3 trials, n = 571), neoadjuvant CT plus surgery versus neoadjuvant RT plus surgery (2 trials, n = 179), neoadjuvant RT plus surgery versus surgery (2 trials, n = 150), neoadjuvant RT plus surgery versus RT (2 trials, n = 379), neoadjuvant CCRT plus surgery versus CCRT (2 trials, n = 272), CCRT plus adjuvant CT versus CCRT (3 trials, n = 1,237), and CCRT plus adjuvant CT versus RT plus adjuvant CT (2 trials, n = 505). Three-year PFS was available for direct comparison in the following comparisons: neoadjuvant CT plus surgery versus surgery (4 trials, n = 605), neoadjuvant CT plus surgery versus neoadjuvant RT plus surgery (2 trials, n = 179), neoadjuvant RT plus surgery versus surgery (2 trials, n = 150), and CCRT plus adjuvant CT versus CCRT (3 trials, n = 1,654). However, none of the direct comparisons for both OS and PFS were significant. Forrest plots for pairwise treatment comparisons are presented in **Figure S1**.

### Network Meta-Analysis

The results of pooled estimates of 3-year OS and PFS are summarized in **Table 2**. In terms of 3-year OS, one primary outcome, was evaluated in 23 trials. CCRT showed a significant advantage over RT alone (OR, 0.56; 95% CI, 0.33–0.94), surgery alone (OR, 0.53; 95% CI, 0.28–0.98), and neoadjuvant RT plus surgery (OR, 0.52; 95% CI, 0.28–0.99). Neoadjuvant CT plus CCRT was significantly inferior to CCRT (OR, 0.23; 95% CI, 0.07–0.75) and CCRT plus adjuvant CT (OR, 0.28; 95% CI, 0.08–0.98). As to 3-year PFS, the treatments of neoadjuvant CCRT plus surgery (OR, 3.01; 95% CI, 1.03–8.83), neoadjuvant CT plus CCRT (OR, 3.69; 95% CI, 1.18–11.58), neoadjuvant CT plus surgery (OR, 4.73; 95% CI, 1.04–21.51), surgery alone (OR, 5.56; 95% CI, 1.19–25.96), neoadjuvant RT plus surgery (OR, 6.15; 95% CI, 1.58–24.01), and RT alone (OR, 8.47; 95% CI, 1.84–38.96) were all inferior to CCRT plus adjuvant CT. Moreover, the neoadjuvant RT plus surgery (OR, 3.69; 95% CI, 1.03–13.25) and RT alone (OR, 5.08; 95% CI, 1.19–21.70) were also inferior to CCRT. Interestingly, the 3-year PFS of CCRT plus adjuvant CT was statistically significantly better than that of CCRT (OR, 0.60; 95% CI, 0.38–0.96).

**Table 2:** Pooled estimates for the overall survival and progression free survival.

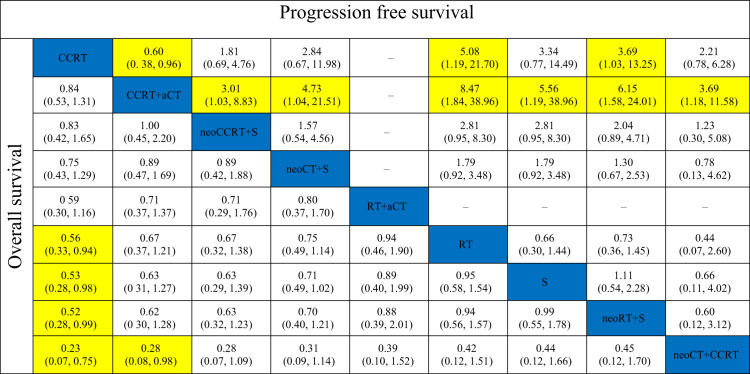

a, adjuvant; neo, neoadjuvant; CCRT, chemoradiotherapy; CT, chemotherapy; RT, radiotherapy; S, surgery. Highlighted boxes indicate the significant odds ratio (95% confidence interval) of the corresponding pairs.

**Figure 3:**
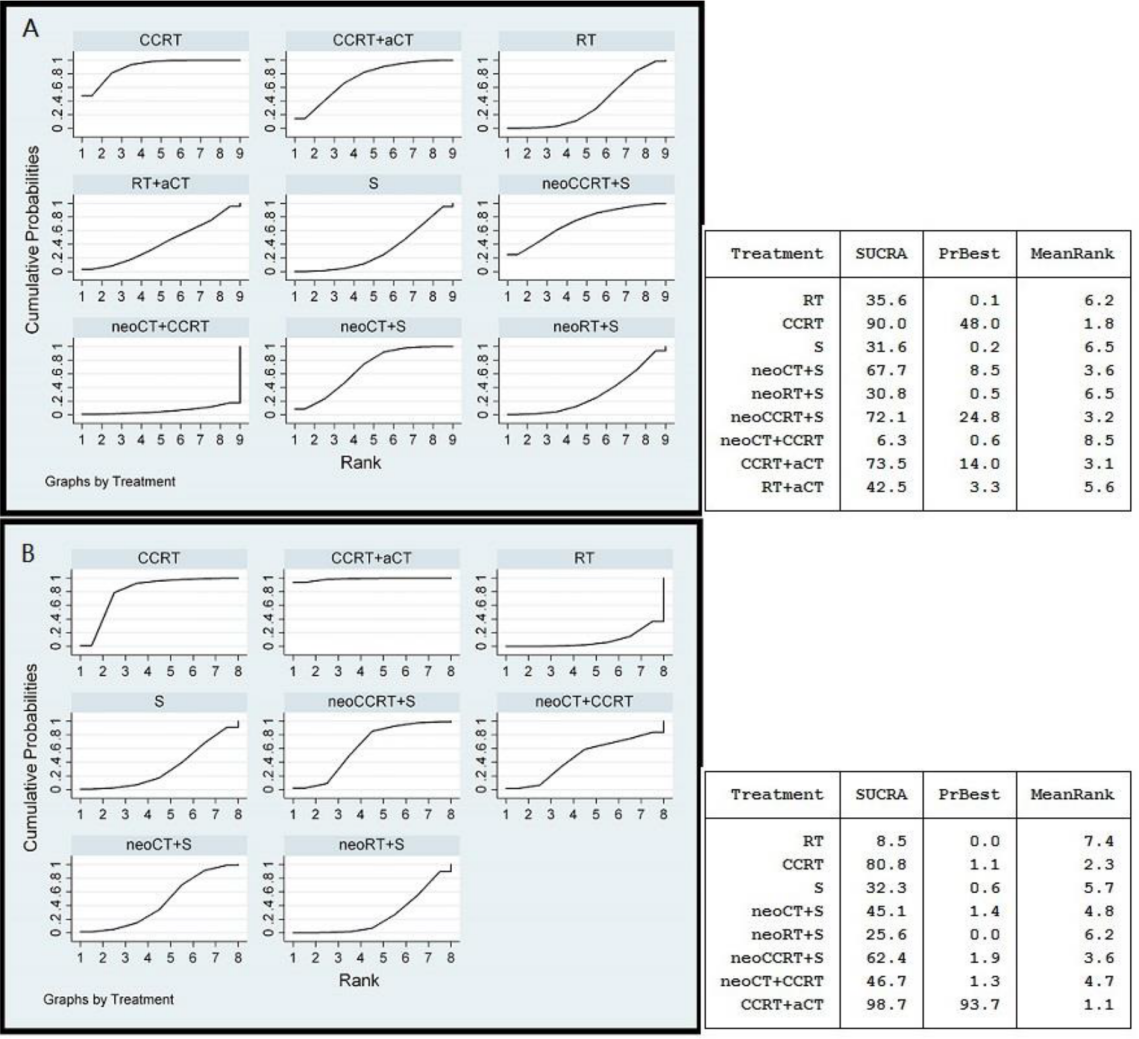
Ranking curves for the overall survival **(A)** and progression free survival **(B)** using random effects model. The rankings have been based on the surface under the cumulative ranking (SUCRA) values with the best rank obtained by the modality with the highest SUCRA value. a, adjuvant; neo, neoadjuvant; CCRT, chemoradiotherapy; CT, chemotherapy; RT, radiotherapy; S, surgery.

The results of SUCRA indicated that CCRT and CCRT plus adjuvant CT were likely to be the most optimal strategies for LACC in terms of OS and PFS (**Figure 3**). As shown in **Figure 3A**, CCRT (0.900) and CCRT plus adjuvant CT (0.735) had the highest probability to represent the most effective treatment approaches for LACC, to be the best and second best therapeutic options, respectively. The other treatments were ranked in descending order as follows: neoadjuvant CCRT plus surgery (0.721), neoadjuvant CT plus surgery (0.677), RT followed by adjuvant CT (0.425), RT alone (0.356), surgery alone (0.316), neoadjuvant RT plus surgery (0.308), and neoadjuvant CT plus CCRT (0.063). **Figure 3B** shows that rank for PFS in descending order: CCRT plus adjuvant CT (0.987), CCRT (0.808), neoadjuvant CCRT plus surgery (0.624), neoadjuvant CT plus CCRT (0.467), neoadjuvant CT plus surgery (0.451), surgery alone (0.323), neoadjuvant RT plus surgery (0.256) and RT alone (0.085).

Analyses were also conducted to group neoadjuvant therapies before surgery together and adjuvant CT after CCRT or RT together and compared with CCRT alone. PFS advantages were found for adjuvant CT after CCRT/RT compared with CCRT (OR, 0.60; 95% CI, 0.38–0.96) and neoadjuvant therapies before surgery (OR, 0.33; 95% CI, 0.11–0.97). However, no OS advantage was found for the same comparison groups (**Figure S2**).

### Consistency Test and Publication Bias

The included trials were demonstrated to be of high quality according to Jadad scale (**Table S1**). The NMA was high reliable because no evidence of inconsistency among most comparisons was found by node-splitting method. We also did not find significant publication bias from the results of Begg’s test (**Figure S3**).

## Discussion

To our knowledge, this study was the first one to compare all commonly practiced treatment modalities for LACC. We integrated both the direct and indirect comparisons by employing a Bayesian NMA, remedying the insufficiency of traditional meta-analysis. We found that, among nine modalities, CCRT and CCRT plus adjuvant CT were likely to be the optimal strategies for LACC in terms of both 3-year OS and PFS.

Neoadjuvant treatments have the potential to eradicate micrometastases and could reduce systemic failures, in addition to facilitating local control by surgical resection. Neoadjuvant treatments also increase radio-sensitivity and decrease the hypoxic cell fraction. A recent review did not find sufficient evidence that neoadjuvant treatments followed by surgery improved the survival of LACC when compared with patients who were treated with RT or CCRT alone (47). It also failed to distinguish the modality of neoadjuvant treatments, i.e., CT, RT and CCRT. In our NMA, a total of 18 randomized controlled trials about neoadjuvant treatments (i.e., neoadjuvant CT plus surgery, neoadjuvant CT plus CCRT, neoadjuvant RT plus surgery and neoadjuvant CCRT plus surgery) were covered. Still, none of the neoadjuvant treatment outcomes surpassed that of CCRT alone.

In contrast to previous meta-analysis (11), the risk reduction of death associated with neoadjuvant CT was not statistically significant in our NMA. The previous meta-analysis did not include 3 randomized controlled trials by Wen et al. (39), Li et al. (40), and Yang et al. (46). In addition, none of the previous meta-analyses compared neoadjuvant CT followed by surgery with CCRT, the standard treatment modality. There was only one head-to-head phase III clinical trial (29), in which Gupta et al. found it was no difference in OS between these two strategies; however, CCRT resulted in superior disease-free survival in stage IIB disease. Neoadjuvant CT before CCRT was also evaluated in the prospective randomized phase II trial (21), which suggests that the addition of neoadjuvant CT to CCRT is associated with an inferior PFS and a lower OS when compared with CCRT. Nevertheless, the result of one ongoing head-to-head phase III clinical trial evaluating the role of neoadjuvant CT plus CCRT will be of interest (ClinicalTrials.gov identifier: NCT01566240). Although no improvement in survival was found when compared with surgery alone, neoadjuvant CT (48), neoadjuvant RT (37), and neoadjuvant CCRT (31, 32) before surgery are also used in reality and clinical studies for LACC.

A meta-analysis found adjuvant CT after CCRT may be beneficial because 35% of patients experience disease progression after CCRT alone (49). Although many prospective phase II studies show an increased response rate with adjuvant CT after CCRT with high 80–90% survival rates (50–52), there is still much debate when compared with CCRT. Two randomized controlled trials showed increased PFS or OS using CCRT plus adjuvant CT (16, 17) whereas two other trials could not demonstrate such a benefit (15, 18). Overall, our summary analysis suggested that adjuvant CT after CCRT bring a significant PFS but no OS advantage compared with CCRT. Our SUCRA displayed that CCRT plus adjuvant CT was the best therapeutic options in terms of 3-year PFS. The interesting findings could be explained by that the adjuvant CT after CCRT eradicated the micrometastases that may have not be eradicated by CCRT. The adjuvant CT could also consolidate the local effects of the concomitant chemo(brachy)radiotherapy (17). Thus, adjuvant CT after CCRT could be beneficial for certain patient groups. However, toxicities were more frequent in the adjuvant CT after CCRT group. For example, in the phase III trial, grade 3 and 4 toxicities were 86.5% for adjuvant CT after CCRT arm, but 46.3% for CCRT arm (*P <*0.001) (16). However, in this trial, a possible selective benefit of adjuvant CT on a more advanced stage or certain histopathology (adenocarcinoma in particular) might be diluted by including stage II patients or squamous cell carcinoma, and they comprised the majority cervical cancer population and these diseases could be managed by CCRT alone. In line with this, Dueñas-González et al. (16) also found greater benefit of adjuvant CT after CCRT were observed in stages III–IV or for adenocarcinoma. Therefore, phase III trials specifically targeted for specific disease populations such as stage III–IV disease and those with adenocarcinoma are needed to elucidate whether adjuvant CT after CCRT is better than CCRT under certain circumstances.

Radical hysterectomy with pelvic lymphadenectomy has been a treatment option for LACC (category 2B) (53). In our study, CCRT showed a significant PFS and OS advantage over surgery alone by indirect comparison. Currently, there are no prospective randomized controlled trials directly investigating surgery in compared with CCRT for LACC. Since 20-year OS of RT group and surgery group were 77 and 72% (*P* = 0.280) (54) and the National Cancer Institute suggested that CCRT should replace RT alone for LACC (10), direct comparison of surgery and CCRT might not be necessary. Thus, in general, our results still support that CCRT is the treatment of choice for LACC. The refinement of CCRT regimens should be considered as the direction for future research.

Our conclusion is strengthened by the Bayesian NMA used. Firstly, this study pooled together direct and indirect evidence within comprehensive comparisons. Secondly, we identified regimens that were superior to others, which made interpretation straightforward from a clinical point of view. The limitations of this NMA also need to be acknowledged. Firstly, we did not have access to individual patient data, which limited the precision of our estimates. Secondly, the characteristics of the included studies confined the quality of our analysis. Although transitivity assumption was met, there was no accepted method to test similarity and subjective judgments can only be made by comparing clinical studies characteristics. Thirdly, the CT, RT, CCRT and surgery techniques and regimens also differ. The studies were reported over a span of 32 years (1987–2019). Thus, the availability of the treatment facilities and technology over the years should be very much different (e.g., RT planning and delivery, CT drugs and FIGO staging). Nevertheless, the current NMA is not a substitute for direct head-to-head comparison trials, but suggests which treatment modality may represent the most appropriate for further evaluation in future studies.

In conclusion, our Bayesian NMA supports CCRT as the standard therapy for LACC. Compared with CCRT, although CCRT plus adjuvant CT has shown a PFS benefit for LACC, future studies needed to find an appropriate chemotherapy regimen which improved OS in some patient groups. Therefore, in general, CCRT is still the treatment of choice for LACC. Current and future research should be focused on developing the most effective CCRT and CCRT plus adjuvant CT regimens.

## Data Availability Statement

The original contributions presented in the study are included in the article/**Supplementary Material**. Further inquiries can be directed to the corresponding author.

## Author Contributions

BP and YQ designed the study. YQ and HL completed the data collection. YQ and BP drafted the manuscript. BP provided clinical insights and did the literature review and help with the drafting of the manuscript. All authors listed have made a substantial, direct, and intellectual contribution to the work and approved it for publication.

## Conflict of Interest

The authors declare that the research was conducted in the absence of any commercial or financial relationships that could be construed as a potential conflict of interest.

## Publisher’s Note

All claims expressed in this article are solely those of the authors and do not necessarily represent those of their affiliated organizations, or those of the publisher, the editors and the reviewers. Any product that may be evaluated in this article, or claim that may be made by its manufacturer, is not guaranteed or endorsed by the publisher.
