# Supplementary material for: Neoadjuvant and Adjuvant Treatments Compared to Concurrent Chemoradiotherapy for Patients With Locally Advanced Cervical Cancer: A Bayesian Network Meta-Analysis
Source: Front Oncol. 2022 Mar 16;12:745522. doi: 10.3389/fonc.2022.745522 (PMC8966774; doi:10.3389/fonc.2022.745522)
Supplement: Supplementary file 1 [file DataSheet_1.pdf]

Table S1. Jadad Scale for 24 Included Studies

Author	Blinding	Randomization	An account of patients	High quality
Lorvidhay	0	2	1	√
Duenas	0	2	1	√
Tang	0	2	1	√
Tangjitgamol	0	2	1	√
Costa	0	2	1	√
Gupta	0	2	1	√
Wang	0	2	1	√
Morice	0	2	1	√
Getina	0	2	1	√
Keys	0	2	1	√
Chang	0	2	1	√
Benedetti	0	2	1	√
Perez	0	2	1	√
Keys	0	2	1	√
Yamauchi	0	2	1	√
Landoni	0	2	1	√
Wen	0	2	1	√
Li	0	2	1	√
Sardi	0	2	1	√
Cai	0	2	1	√
Eddy	0	2	1	√
Chen	0	2	1	√
Katsumata	0	2	1	√
Yang	0	2	1	√

* When total score≥3, the relative RCT will be considered as of high quality.

The questions were as follows: 1. Was the study described as randomized? 2. Was the study described as double blind? 3. Was there a description of withdrawals and dropouts? To receive the corresponding point, an article should describe the number of withdrawals and dropouts, in each of the study groups, and the underlying reasons. Additional points were given if: 1. The method of randomization was described in the paper, and that method was appropriate. (1 extra point in randomization part); 2. The method of blinding was described, and it was appropriate. (1 extra point in blinding part)

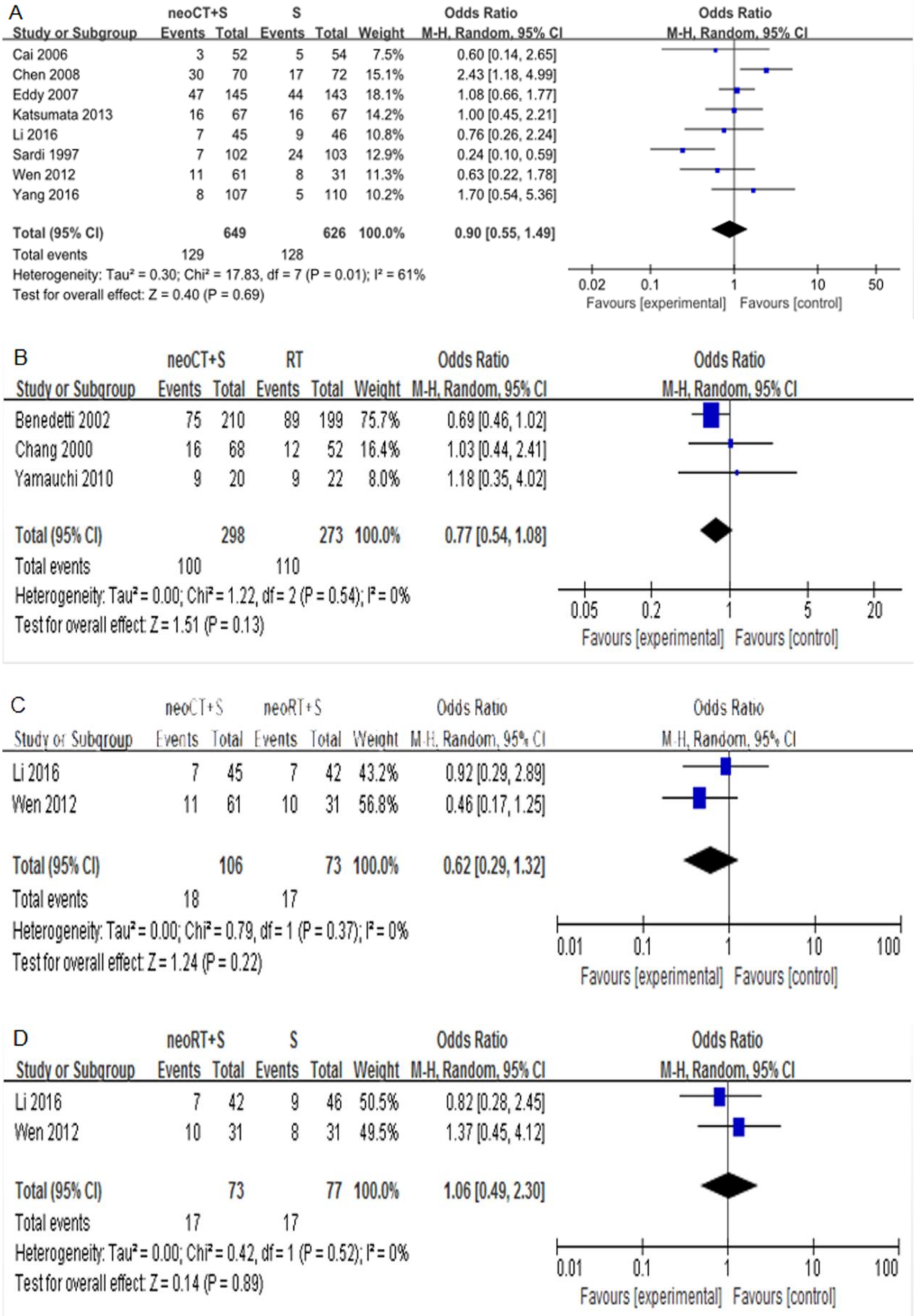
A
neoCT+S
S
Odds Ratio
Odds Ratio
Study or Subgroup Events Total Events Total Weight M-H, Random, 95% CI M-H, Random, 95% CI
Cai 2006 3 52 5 54 7.5% 0.60 [0.14, 2.65]
Chen 2008 30 70 17 72 15.1% 2.43 [1.18, 4.99]
Eddy 2007 47 145 44 143 18.1% 1.08 [0.66, 1.77]
Katsumata 2013 16 67 16 67 14.2% 1.00 [0.45, 2.21]
Li 2016 7 45 9 46 10.8% 0.76 [0.26, 2.24]
Sardi 1997 7 102 24 103 12.9% 0.24 [0.10, 0.59]
Wen 2012 11 61 8 31 11.3% 0.63 [0.22, 1.78]
Yang 2016 8 107 5 110 10.2% 1.70 [0.54, 5.36]
Total (95% CI) 649 626 100.0% 0.90 [0.55, 1.49]
Total events 129 128
Heterogeneity: Tau² = 0.30; Chi² = 17.83, df = 7 (P = 0.01); I² = 61%
Test for overall effect: Z = 0.40 (P = 0.69)
0.02 0.1 1 10 50
Favours [experimental] Favours [control]
B
neoCT+S
RT
Odds Ratio
Odds Ratio
Study or Subgroup Events Total Events Total Weight M-H, Random, 95% CI M-H, Random, 95% CI
Benedetti 2002 75 210 89 199 75.7% 0.69 [0.46, 1.02]
Chang 2000 16 68 12 52 16.4% 1.03 [0.44, 2.41]
Yamauchi 2010 9 20 9 22 8.0% 1.18 [0.35, 4.02]
Total (95% CI) 298 273 100.0% 0.77 [0.54, 1.08]
Total events 100 110
Heterogeneity: Tau² = 0.00; Chi² = 1.22, df = 2 (P = 0.54); I² = 0%
Test for overall effect: Z = 1.51 (P = 0.13)
0.05 0.2 1 5 20
Favours [experimental] Favours [control]
C
neoCT+S
neoRT+S
Odds Ratio
Odds Ratio
Study or Subgroup Events Total Events Total Weight M-H, Random, 95% CI M-H, Random, 95% CI
Li 2016 7 45 7 42 43.2% 0.92 [0.29, 2.89]
Wen 2012 11 61 10 31 56.8% 0.46 [0.17, 1.25]
Total (95% CI) 106 73 100.0% 0.62 [0.29, 1.32]
Total events 18 17
Heterogeneity: Tau² = 0.00; Chi² = 0.79, df = 1 (P = 0.37); I² = 0%
Test for overall effect: Z = 1.24 (P = 0.22)
0.01 0.1 1 10 100
Favours [experimental] Favours [control]
D
neoRT+S
S
Odds Ratio
Odds Ratio
Study or Subgroup Events Total Events Total Weight M-H, Random, 95% CI M-H, Random, 95% CI
Li 2016 7 42 9 46 50.5% 0.82 [0.28, 2.45]
Wen 2012 10 31 8 31 49.5% 1.37 [0.45, 4.12]
Total (95% CI) 73 77 100.0% 1.06 [0.49, 2.30]
Total events 17 17
Heterogeneity: Tau² = 0.00; Chi² = 0.42, df = 1 (P = 0.52); I² = 0%
Test for overall effect: Z = 0.14 (P = 0.89)
0.01 0.1 1 10 100
Favours [experimental] Favours [control]

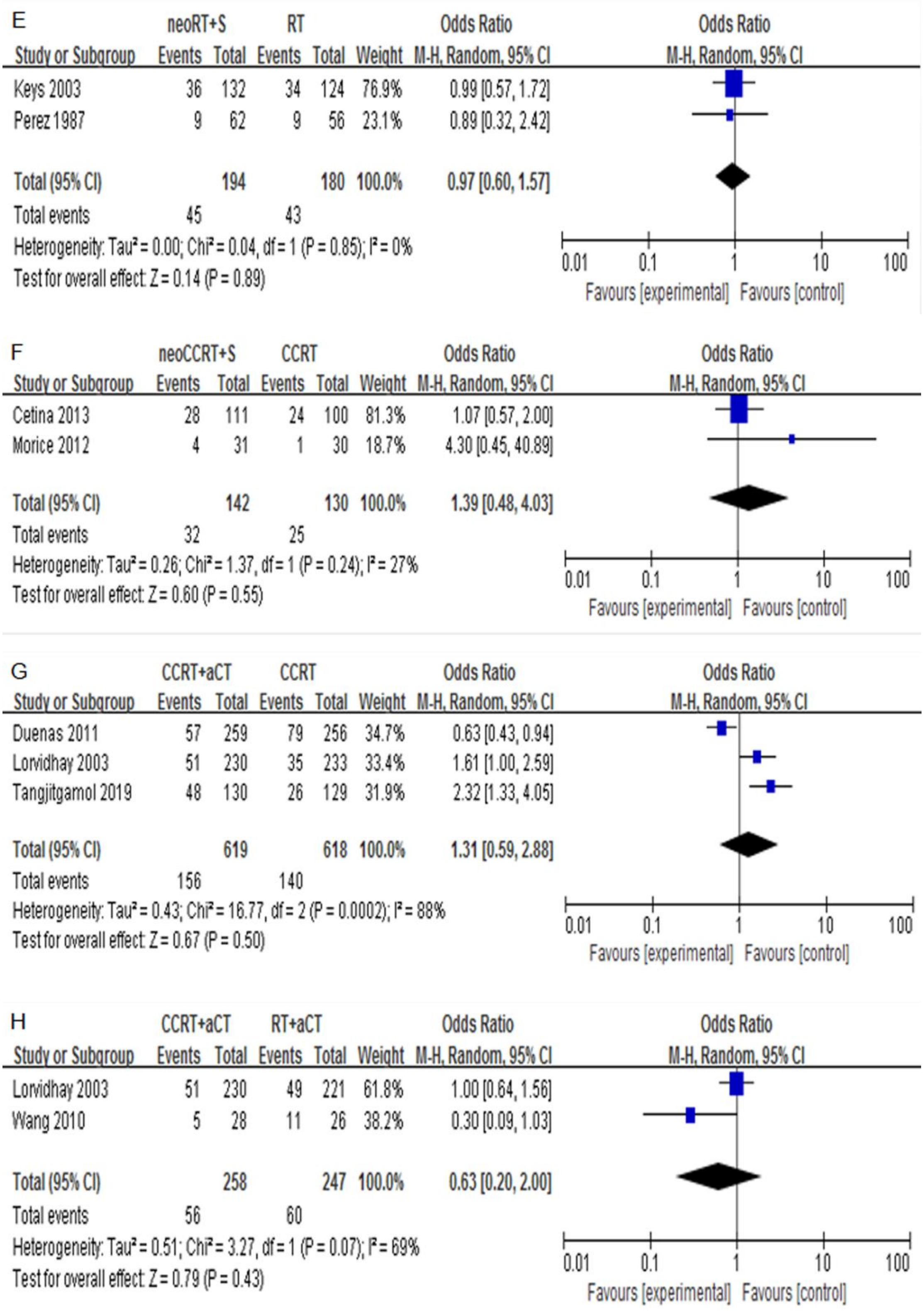
E
neoRT+S
RT
Odds Ratio
Odds Ratio
Study or Subgroup Events Total Events Total Weight M-H, Random, 95% CI
M-H, Random, 95% CI
Keys 2003 36 132 34 124 76.9% 0.99 [0.57, 1.72]
Perez 1987 9 62 9 56 23.1% 0.89 [0.32, 2.42]
Total (95% CI) 194 180 100.0% 0.97 [0.60, 1.57]
Total events 45 43
Heterogeneity: Tau² = 0.00; Chi² = 0.04, df = 1 (P = 0.85); I² = 0%
Test for overall effect: Z = 0.14 (P = 0.89)
0.01 0.1 1 10 100
Favours [experimental] Favours [control]
F
neoCCRT+S
CCRT
Odds Ratio
Odds Ratio
Study or Subgroup Events Total Events Total Weight M-H, Random, 95% CI
M-H, Random, 95% CI
Cetina 2013 28 111 24 100 81.3% 1.07 [0.57, 2.00]
Morice 2012 4 31 1 30 18.7% 4.30 [0.45, 40.89]
Total (95% CI) 142 130 100.0% 1.39 [0.48, 4.03]
Total events 32 25
Heterogeneity: Tau² = 0.26; Chi² = 1.37, df = 1 (P = 0.24); I² = 27%
Test for overall effect: Z = 0.60 (P = 0.55)
0.01 0.1 1 10 100
Favours [experimental] Favours [control]
G
CCRT+aCT
CCRT
Odds Ratio
Odds Ratio
Study or Subgroup Events Total Events Total Weight M-H, Random, 95% CI
M-H, Random, 95% CI
Duenas 2011 57 259 79 256 34.7% 0.63 [0.43, 0.94]
Lorvidhay 2003 51 230 35 233 33.4% 1.61 [1.00, 2.59]
Tangjitgamol 2019 48 130 26 129 31.9% 2.32 [1.33, 4.05]
Total (95% CI) 619 618 100.0% 1.31 [0.59, 2.88]
Total events 156 140
Heterogeneity: Tau² = 0.43; Chi² = 16.77, df = 2 (P = 0.0002); I² = 88%
Test for overall effect: Z = 0.67 (P = 0.50)
0.01 0.1 1 10 100
Favours [experimental] Favours [control]
H
CCRT+aCT
RT+aCT
Odds Ratio
Odds Ratio
Study or Subgroup Events Total Events Total Weight M-H, Random, 95% CI
M-H, Random, 95% CI
Lorvidhay 2003 51 230 49 221 61.8% 1.00 [0.64, 1.56]
Wang 2010 5 28 11 26 38.2% 0.30 [0.09, 1.03]
Total (95% CI) 258 247 100.0% 0.63 [0.20, 2.00]
Total events 56 60
Heterogeneity: Tau² = 0.51; Chi² = 3.27, df = 1 (P = 0.07); I² = 69%
Test for overall effect: Z = 0.79 (P = 0.43)
0.01 0.1 1 10 100
Favours [experimental] Favours [control]

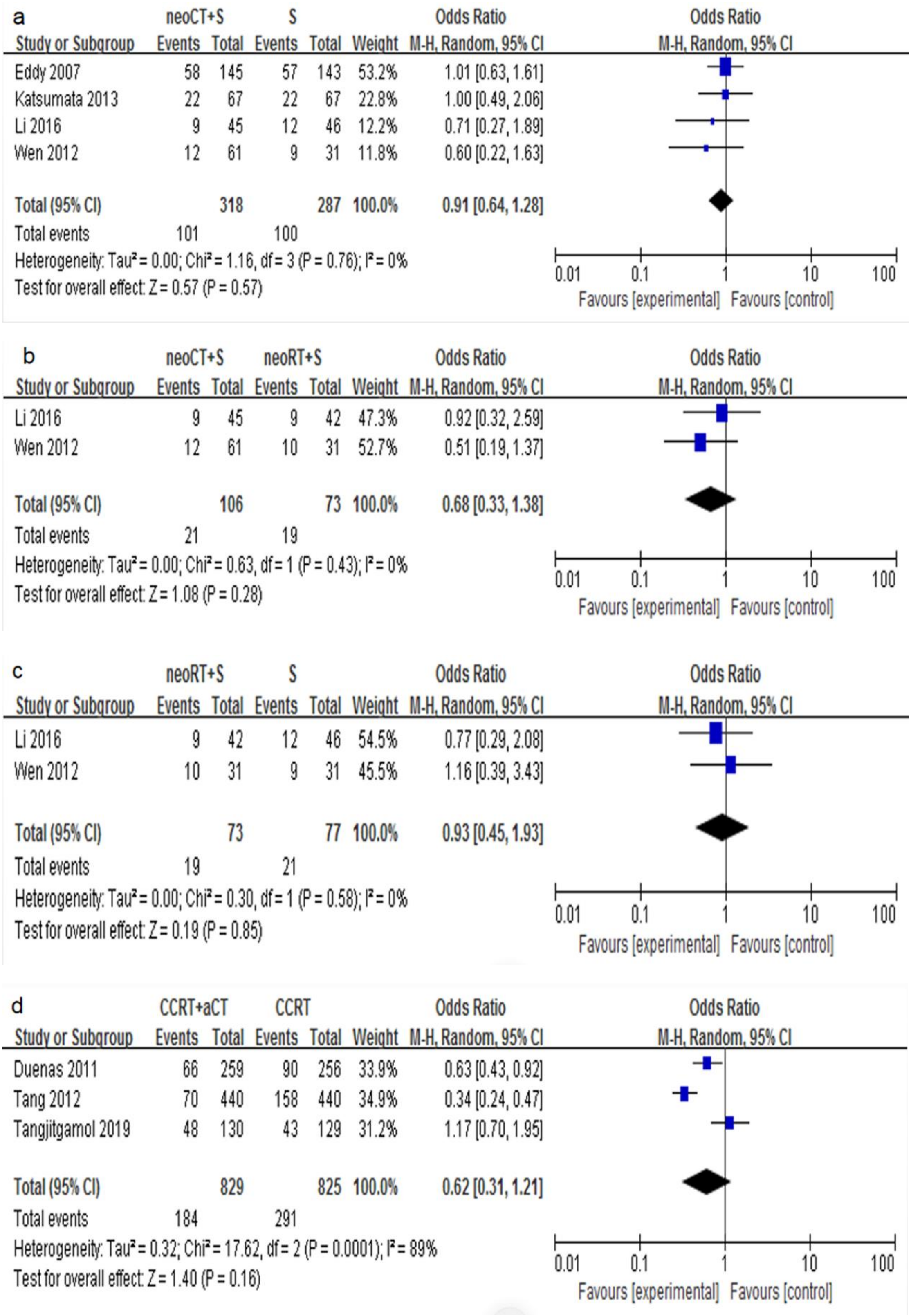

Supplementary Figure 1. Forest plots for the overall survival (A, B, C, D, E, F, G, H) and progression free survival (a, b, c, d).

Abbreviations: a, adjuvant; neo, neoadjuvant; CCRT, chemoradiotherapy; CT,

chemotherapy; RT, radiotherapy; S, surgery.

Progression free survival

Overall survival

CCRT	**0.60 (0.38, 0.96)**	1.81 (0.69, 4.74)	2.87 (0.93, 8.87)	2.02 (0.68, 5.99)	2.87 (0.93, 8.87)
0.81 (0.52, 1.26)	CCRT/RT+aCT	**3.01 (1.03, 8.80)**	**4.78 (1.41, 16.24)**	**3.37 (1.03, 10.98)**	**3.69 (1.18, 11.54)**
0.76 (0.47, 1.21)	0.94 (0.52, 1.68)	Neo+S	1.59 (0.88, 2.85)	1.12 (0.68, 1.84)	1.59 (0.88, 2.85)
0.63 (0.38, 1.03)	0.78 (0.44, 1.38)	0.83 (0.57, 1.20)	RT	0.70 (0.33, 1.52)	0.77 (0.17, 3.58)
0.56 (0.31, 1.00)	0.69 (0.36, 1.35)	0.74 (0.52, 1.06)	0.89 (0.56, 1.43)	S	1.10 (0.24, 4.93)
**0.23 (0.07, 0.75)**	0.29 (0.08, 1.01)	0.31 (0.09, 1.08)	0.37 (0.11, 1.32)	0.42 (0.11, 1.53)	NeoCT+CCRT

Supplementary Figure 2. Pooled estimates for the overall survival and progression free survival. Highlighted boxes indicate the significant odds ratio (95% confidence interval) of the corresponding pairs.

Abbreviations: a, adjuvant; Neo, neoadjuvant; CCRT, chemoradiotherapy; CT, chemotherapy; RT, radiotherapy; S, surgery.

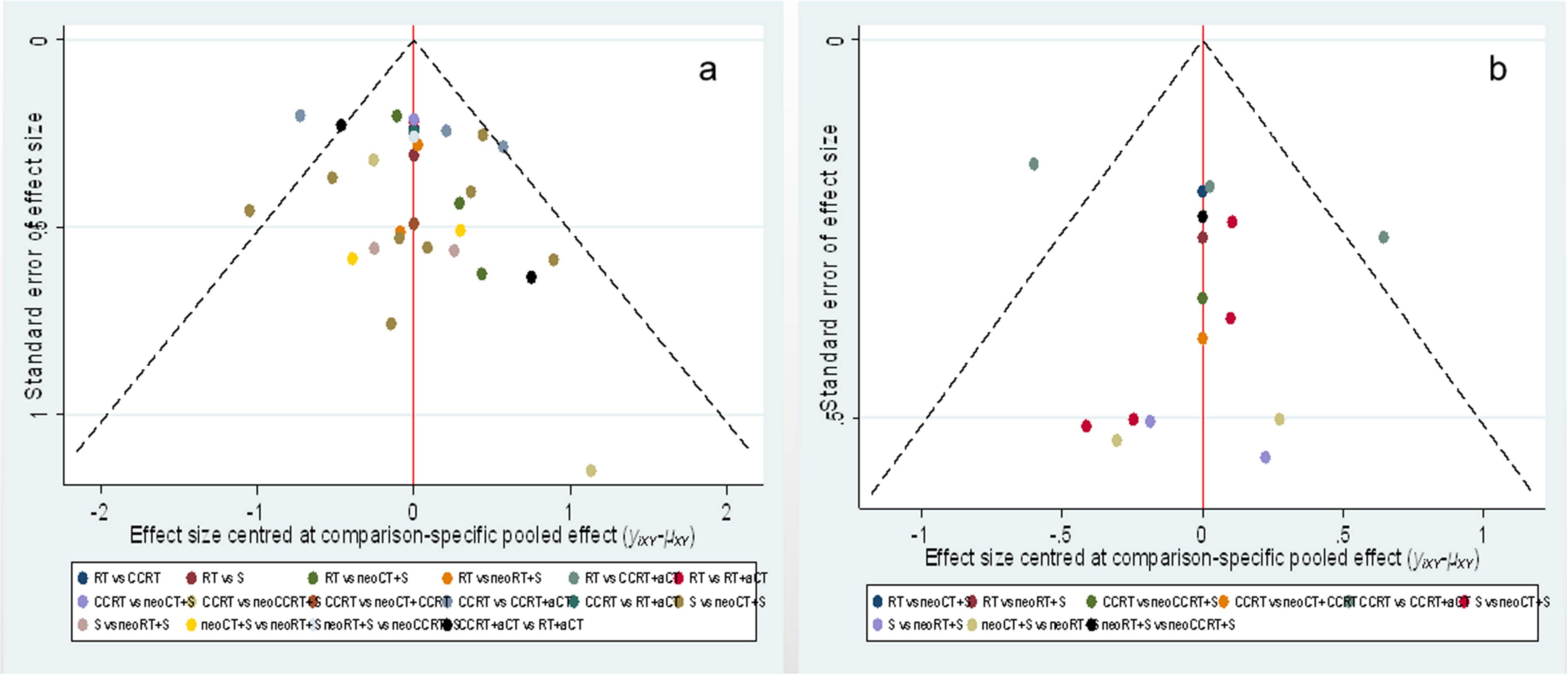

Supplementary Figure 3. Funnel plot for risk of publication bias for the overall survival (a) and progression free survival (b).

Abbreviations:a, adjuvant; neo, neoadjuvant; CCRT, chemoradiotherapy; CT, chemotherapy; RT, radiotherapy; S, surgery.
